# An Explanatory Framework for Understanding Mental Health Needs of Afghan Refugees Resettled in the Global North

**DOI:** 10.1002/ijop.70117

**Published:** 2025-10-03

**Authors:** Savannah Dieste, Freschta Naseri, John Dykema, Chaundra Merrell, Alexander Laywell, Hamayoun Kargar, Sunita M. Stewart

**Affiliations:** ^1^ Department of Psychiatry University of Texas Southwestern Medical Center Dallas Texas USA; ^2^ International Rescue Committee Dallas Texas USA; ^3^ Division of Psychology University of Texas Southwestern Medical Center Dallas Texas USA; ^4^ Department of Psychiatry Children's Health Children's Medical Center Dallas Texas USA

**Keywords:** Afghanistan, clinical recommendations, explanatory models, refugee mental health, underserved groups

## Abstract

Afghans are the second‐largest refugee population in the world, yet their mental health needs are understudied. This qualitative investigation has two aims: (1) to examine the mental health beliefs of Afghan refugees in one ‘Global North’ nation who fled following the 2021 Taliban takeover and (2) to provide an example of how explanatory models might be used to support mental health care in diverse populations. Participants (*N* = 21) were Afghan refugees who resettled in the US after 2021 and cultural experts. We conducted semi‐structured interviews to uncover the mental health belief framework of this population by exploring explanatory domains: ‘*experiences of distress*’, ‘*causal attributions for suffering*’, ‘*coping strategies*’ and ‘*goals for wellbeing*’. Also explored were ‘*attitudes towards host culture treatment*’. Thematic analysis revealed several key findings: emotional suffering is ubiquitous and not a target for treatment; distress is caused by specific circumstances and loss, primary coping strategies are avoidance and reliance on family, community, and faith, and primary goals are survival and functioning in valued roles. ‘*Recommended clinical approaches*’ for host culture practitioners that bridge provider and client models are discussed, which offer tools to develop rapport, allow for cultural adaptation and increase acceptability of treatments offered.

## 1 Introduction

### 1.1 Explanatory Frameworks

The term ‘explanatory models of illness’ describes culturally based beliefs about misfortune, suffering, health and illness (Kleinman et al. 1978). These beliefs include attributions for the cause, the course and conditions that effectively reduce the symptoms experienced (Dinos et al. 2017). These beliefs are multifaceted, fluid and influenced by migration and interaction with clinicians (Dinos et al. 2017).

That these beliefs contribute to disparities in care is well recognised in mental health service delivery (Dinos et al. 2017). Because mainstream mental health and evidence‐based practices were not developed with the needs of ethnic minorities in mind, there can be a mismatch between client and practitioner beliefs about illness, health and treatment (Burgess et al. 2004). This disconnect often discourages ethnic minorities from seeking and staying in treatment (McGuire and Miranda 2008).

Consistent with this framework, clients are most satisfied when they share an understanding of suffering and healing with their practitioner (Callan and Littlewood 1998). Client satisfaction is positively correlated with treatment adherence and positive outcomes (Barbosa et al. 2012). Clinicians can leverage their understanding of client health beliefs to promote treatment acceptability, build rapport and culturally adapt evidence‐based practices (Bhui and Bhugra 2002). Clinicians working with refugee populations who demonstrate cultural competence (i.e., the ability to provide care to diverse patients and meet their cultural needs) and cultural humility (i.e., the initiative to self‐reflect, correct power imbalances and partner with cultural experts) will provide more ethical and effective mental health services (Lau and Rodgers 2021).

To this end, several tools have been developed to assess the health beliefs of culturally diverse patients in clinical settings. Among them is Bart's Explanatory Model Inventory (BEMI; Rüdell et al. 2009), which offers a framework to elicit culturally variable perceptions of illness. The model identifies domains of enquiry that would aid in this process: experience of symptoms (overlapping with consequences of symptoms), cause/aetiology, course of illness, and treatment/management of distress.

### 1.2 Afghan Refugee Populations

Afghans are the second‐largest refugee population in the world, largely attributed to continuous war and political unrest spanning generations (UNHCR 2023). Recent conflicts include decades of civil war, followed by the United States' invasion of Afghanistan from 2001 to 2021. Violence, natural disasters and economic turmoil have compounded with war‐related intergenerational trauma for many (IRC 2023). This has resulted in an exodus of refugees from Afghanistan to various nations.

As of 2020, nations in the ‘Global North’^1^ who share a broadly common approach to mental wellness care hosting the largest number of Afghan refugees included Germany (148,000), Austria (40,000), France (32,000) and Sweden (30,000) (Buchholz 2021). Since the US military and other international forces withdrew from Afghanistan and the Taliban abruptly took over the country in 2021, roughly 100,000 Afghans have arrived in the United States, with many still seeking entry (US DoS 2023). Canada has also welcomed over 40,000 new arrivals since 2021 (Immigration, Refugees and Citizenship Canada 2024). This population of refugees is heterogeneous in terms of ethnicity, SES, religion and conservativism, creating variability in acculturation (i.e., conservativism and acceptance of the norms of their host culture) and resettlement demands. Our study is focused on this population of Afghan refugees who migrated following the 2021 Taliban takeover, whose beliefs and needs likely differ from past populations of Afghan refugees (Brea Larios et al. 2022).

Each nation has nuanced policies regarding the status of asylum seekers and refugees, which can induce uncertainty about longer‐term status. Many Afghan refugees displaced by the 2021 regime change faced peril due to their employment with foreign governments. For example, roughly 40% of this population of Afghan refugees in the United States are on special immigrant visas (‘SIVs’) (US DoS 2023), awarded to individuals who worked for the US forces and their spouses and children. However, many could not bring their extended family with them in their sudden and dangerous departure.

### 1.3 Mental Health Needs of Refugees From Afghanistan

Generational trauma, pre‐migration stressors and traumatic aspects of flight are compounded by adaptation demands in host countries (Lipson 1993). Psychological distress among Afghan refugees in the United States has been associated with being female, financial stress, unclear immigration status, social functioning, loneliness, cultural adaptation difficulties, discrimination and low education (Alemi et al. 2015, 2023; Li et al. 2016). Debilitating depressive symptoms among Afghan refugees have been attributed to both pre‐ and post‐migration traumas and stressors (Alemi et al. 2016; Lavdas et al. 2023). Beliefs about the causes of psychological distress and coping strategies have been found to vary by gender, age, generation and migration experience (Brea Larios et al. 2022; Lavdas et al. 2023). Natural coping strategies for managing distress among Afghan refugees have included social and behavioural activation, avoidance and religion (Sulaiman‐Hill and Thompson 2012). Despite recent evidence indicating stress associated with witnessing the 2021 Taliban takeover through social media (Lavdas et al. 2023), there is a gap in understanding the perceived broader mental health needs of the population of Afghan refugees in the United States who were displaced by the 2021 regime change, many of whom may be at higher risk for collective trauma and poor mental health outcomes (Kirsch et al. 2024).

Traditional explanatory models in Afghanistan recognise only severe mental illness and aim to rid evil spirits that cause the illness through seclusion, restraint, food and water restriction, herbal medicine and prayer (van de Put 2002). Afghan communities traditionally attribute distress to economic barriers, which impede valued moral and social aspirations, and rely on their network of family, friends and culture‐specific healers to guide diagnosis and treatment decisions (de Anstiss and Ziaian 2010; Eggerman and Panter‐Brick 2010). Discrepancies between these beliefs and frameworks in the host culture for understanding and treating mental illness might interfere with the acceptability of mainstream treatment and have been understudied (Alemi et al. 2017). Many in need are not severely mentally ill but may find value in treatment to manage their distress. Host culture practitioners who seek to provide care to Afghan refugees displaced by the 2021 regime change would benefit from understanding the extent to which their mental health beliefs are congruent with local models.

### 1.4 The Current Study

This study was a collaboration between academics and a community organisation that serves displaced individuals. Using a qualitative design, this study explored the underlying mental health belief framework of Afghan refugees resettled in the United States following the 2021 Taliban takeover. The primary aim of our study was to provide culture‐specific information (e.g., cultural beliefs, values, social imbalances, healing practices and stigma) about our participants' ‘*experiences of distress*’, ‘*causal attributions for suffering*’ and ‘*coping strategies*’. These constructs are common to many frameworks that seek to assess perceptions of illness (Rüdell et al. 2009). We added an enquiry on ‘*goals for wellbeing*’, which can guide clinicians to integrate their model for healing with outcomes valued by the client. We also examined ‘*attitudes towards host culture treatment*’ to elucidate contrasts between frameworks and to derive ‘*recommended clinical approaches*’. Elucidation of such information will allow for opportunities to increase rapport, cultural adaptations and treatment acceptability. Our second aim was to illustrate an approach for bridging culture‐specific explanatory models that may be adapted and applied more broadly across nations and cultures.

## 2 Methods

### 2.1 Participants

Participants (*N* = 21) consisted of three distinct groups. Most were refugees from Afghanistan who had resettled in an urban area of the United States following the 2021 Taliban takeover and were receiving mental health care coordination at the community agency in (deleted for review) (‘RA,’ *n* = 12). The second group was White, US‐born, non‐refugee providers at the community agency who served as cultural experts (‘CE,’ *n* = 4). Although limited by not being of Afghan heritage, CEs were mental health professionals who had worked closely with refugees from Afghanistan and could share their insights and perceptions about typical presentations, responses to what was offered as treatment and the barriers to their care. The third group was Afghan refugees who had resettled in the United States before 2021, whose continued and extensive contact with the local refugee community as interpreters and facilitators allowed them to serve as cultural brokers (‘CE/RA’; *n* = 5). Potential participants were identified by agency staff who obtained permission for the research team to call. No further information was exchanged between the agency staff and the research team. Individuals who were interested in participating and spoke English, Dari or Pashto were scheduled for consent/interviews. Written informed consent was obtained by the interviewer in person before conducting interviews. Our sample was predominantly women (67%) and early middle age (*M* = 36.67, SD = 9.16). Participants were informed that all information shared would be deidentified, and interpreters were bound by agency confidentiality and code of conduct agreements. Ethical approval was obtained by the Institutional Review Board of (deleted for review). RAs and CE/RAs were compensated with a $20 gift card for participating. CEs were not compensated.

### 2.2 Procedures

Semi‐structured interviews were conducted in person at the community agency, on video or by phone between November 2021 and August 2022 until saturation was reached. Interviews averaged 33 min (range: 17–43 min). Language services (Dari and Pashto) were provided by two interpreters (one cisgender man and one cisgender woman) for eight interviewees. Interpreters were community agency employees with formal experience as interpreters who were involved in assisting new arrivals with resettlement. They participated in the study as CE/RAs before interpreting for other participants. All interviews were audio recorded and professionally transcribed. The study team consisted of cisgender individuals (four women and three men), either Afghan American of refugee background, South Asian American of immigrant background or White American.

A semi‐structured interview guide was developed by adapting the BEMI (Rüdell et al. 2009). Our interview guide included questions that addressed three BEMI domains: *Experiences of distress* (e.g., what problems or troubles are you/others facing?), *causal attributions for suffering* (e.g., what caused these problems/troubles?) and *coping strategies* (e.g., what do you do to help yourself feel better?). We also assessed *goals for wellbeing* (e.g., what do you need for your mind to be at ease?). We added a domain to tap *attitudes towards host culture treatment* (e.g., what brings people to the doctor?). Cultural experts were asked modified questions that assessed the same domains. Questions were deliberately worded to minimise jargon and abstract concepts. The interviewer was a White American, cisgender woman on the research team who used the guide flexibly. She was an experienced research assistant pursuing a PhD, with no prior relationships with RA and CE/RA participants and limited interactions with CEs before conducting interviews. Interviews were conducted until we reached saturation.

### 2.3 Data Analysis

Codes were generated inductively by team members using the first two transcripts. The remaining transcripts were coded independently by two coders (one White, US‐born woman and one South Asian‐born woman of immigrant background). Thematic analysis (Braun and Clarke 2006) was used to code interviews. Interrater reliability was calculated as (number of identical codes)/(number of identical codes + number of disparate codes). Agreement was 86% for independently coded transcripts. Disparate codes were resolved before entry into NVivo. Coded data from all transcripts were categorised and clustered, and presented to the broader team to derive inductive themes through a collaborative process with individuals who originated from Afghanistan. The multiple perspectives allowed us to generate interpretations of the data that extended beyond the explanatory framework used to develop hypotheses. We worked as a team to challenge our interpretations and reflect on how our personal experiences might influence perceptions. Input from team members of Afghan refugee background was of the highest importance. These individuals played a key role in refining and extending our observations and interpretations and assisted with writing.

## 3 Results

**FIGURE 1:**
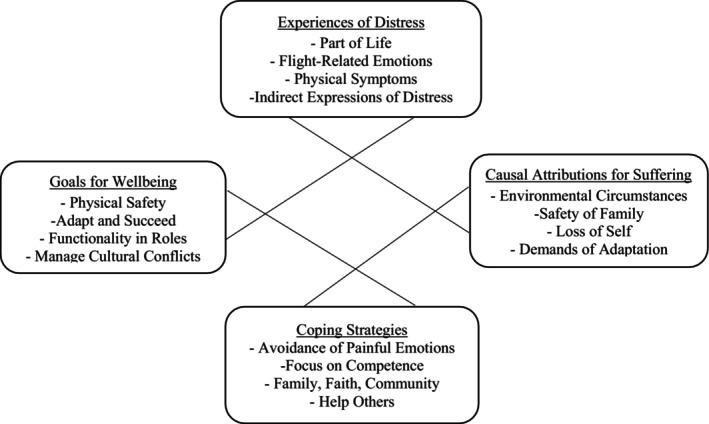
Model linking causal attributions for suffering and goals for wellbeing to experiences and management of distress for participants of Afghan refugee background.

Findings are illustrated below through the voices of participants. Explanatory domains provide an organisational structure to present themes that emerged (bolded and underscored), later presented in Figure 1. Contextual indicators are provided with each quote: refugee from Afghanistan displaced by the 2021 regime change (RA), non‐refugee cultural expert (CE), Afghan‐born cultural expert of refugee background (CE/RA), and woman/man (W/M). Select quotes were edited by the study team to derive the essence of the illustrated point when necessary.

### 3.1 Experiences of Distress

There was a perception that suffering is *part of life*, with some desensitisation to events that cause emotional distress due to their prevalence: ‘People don't know “stress”…that's how they grew up…you just gotta take it…like eating and sleeping…it's just part of life’ (CE/RA, W).

Participants reported expected changes in *emotions related to flight and resettlement*: ‘I think it's a mix of anger and sadness at the same time … and also helpless, like you can't do anything’ (CE/RA, W).

Many were preoccupied by worry about people left behind, particularly separation from family members: ‘We can handle it…but about our relatives now living in Kabul in Afghanistan, we cannot ignore them. Absolutely most of my mind is kept busy with this item’ (RA, W); ‘Even if they give me the whole America. It's still going to be nothing for me if my kids are not here with me’ (RA, W). Some described frank symptoms of trauma expressed through *physical symptoms*: ‘We can't sleep. We can't eat. When we are sleeping, that (nightmare) is still there…high blood pressure…headache always…’ (RA, M); ‘I can't feel one of my feet…I went to the doctor and the doctor said that it's kind of your mental health is affecting that’ (RA, M).

Sometimes the *distress appeared in indirect ways* that were not recognised by the individual:

She was cooking, and she sliced her hand with a knife and almost cut through her thumb…and while she's venting to us about case management issues, she brings up that her mom is in Afghanistan hiding because the Taliban is looking for people who worked at the embassy and she specifically mentioned with the intention of cutting their heads off and kept using that statement…we talked about if the hand injury was a reflection of her mental health status…I think underneath (the anger and frustration) is this deeper feeling of waiting for bad news to come…I think that worry is a distraction and that causes simple things like cutting your hand, but also being hard to work with. (CE, W)

### 3.2 Causal Attributions for Suffering

Most Afghans attributed their distress to being part of their *circumstances or environment*. For many, this began in Afghanistan with generations of war that preceded the chaos of flight: ‘In Afghanistan, nobody was relaxed…Conflict, war, poverty. Lack of resources…’ (RA, M); ‘We're a secular Hazara family which always was in conflict with the religious party of our community…I was never safe in Afghanistan’ (RA, M); ‘When I was trying to come inside the base, they killed seven children in front of me, they shot them, even put the gun on my head too’ (RA, M).

SIV's revealed significant distress concerning their affiliation with the United States *endangering their family*: ‘I myself worked for the (U.S.) and it was a very sensitive department…if someone knew what I was doing, their lives would be in danger. I'm sure they would kidnap them…It was a big (stress)’ (CE/RA, M); ‘My relatives are still in Afghanistan. They are hiding…’ (RA, M).

Post‐migration circumstances posed new challenges such as learning how to live and thrive in the United States and *loss of self* (e.g., identity, status, and agency): ‘We don't know how we can start a job; we don't know how we should handle the license for driving, the asylum process for a green card’ (RA, W); ‘(In) America…I am no one, but in Afghanistan, I was something’ (RA, M); ‘For me it has been a moment of shame…How cruel that I am not able to support myself. I feel like a beggar’ (RA, M).

For most, emotional distress was attributed to *demands of adaptation* and managing the current environment: ‘Things are not moving…things get worse and worse and lots of stress and pressure’ (RA, M). The focus was not on the inner experience of anxiety, worry or sadness; rather on solving problems as they perceive them: ‘There might be some other problem in my life that I don't know…the only thing that I know about us is that we are unemployed’ (RA, W); ‘Once we move to a place or a home or an apartment, I think that most…or all of my problems will be solved’ (RA, M).

### 3.3 Coping Strategies

Some participants reported *dealing with painful emotions by avoiding them*: ‘I have to move on and not even think about it’ (CE/RA, W). For many, the *focus on achieving competence* served not only to distract but also to promote successful adaptation: ‘I do not have time to think negatively. I just barely have time to complete my assignments and get some sleep’ (CE/RA, M).

Stay focused on becoming financially stable and getting those to do lists done…focus in on that…not worry so much and not be inside of their mind as much. (CE, W)

Some had achieved their goals, one step at a time, and derived motivation from confidence achieved at every small step and optimism from past successes: ‘Suppose your income will be one or two dollars per month, but…it has another value…you're feeling confident…this is giving you a motivation…it makes you happy’ (RA, W); ‘Back in Afghanistan we weren't insignificant people, you know – I think right now we just need a little time…we will do much better’ (RA, W).

Those who came to the United States with some resources were better able to harness problem‐solving strategies to find opportunities: ‘My husband and I (searched) online for driver's license, for resume developing, for job searching…we achieved a lot. I obtained my certificate for bookkeeping because my Bachelor's Degree is in economics’ (RA, W).

Consistent with the orientation of Afghan culture, *family* was the first line to provide comfort, companionship and instrumental guidance: ‘Families are like part of the body’ (CE/RA, M); ‘For (those with) family here…it's easy because the family's already helping them and telling them how it works’ (CE/RA, W).

The importance of *faith* as support was also evident: ‘We humans, we do have some reliable sources…whenever we feel bad or sick—mentally sick, we go to them and ask for them for help. The main ones are Allah and then we ask it from our parents’ (RA, M). However, faith could also be a source of stress. Organised religion, such as attendance at a mosque, posed complications because of divisions within Islam, reflected in the heterogeneity of sects in Afghanistan, and also because of differences in religious conservatism within the population. This variability sometimes led to within‐community conflict: ‘One day an Imam asked me why I was not coming to the Mosque…Another asked my girls, why are you not wearing chador (face and body covering). I told him, if you stop my child…I will call the police. It's none of your business’ (RA, M).

But for many, the larger *community* also offered support: ‘Being connected to other women in the community I think is one of the biggest protective factors’ (CE, W); ‘Afghans like to gather on the weekends…the whole week is stressful and at the end of the week they want to get together and have a happy time’ (CE/RA, W).

Several participants discussed their role in *helping others* as a source of wellbeing: ‘I'm helping lots of Afghans because…they can't speak English…that's what makes me feel better that I'm helping someone’ (RA, M); ‘If we have good savings…we can establish a foundation for supporting children in Afghanistan’(RA, W); ‘I'm a live example for (refugees)…I got a job and now I can handle public transportation, open a bank account’ (RA, W).

### 3.4 Goals for Wellbeing

‘Mental health’ or ‘wellbeing’ was not seen as a goal in itself: ‘We definitely talk about happiness and being healthy, but healthy from a physical standpoint, less so from a mental standpoint’ (CE/RA, W). *Physical safety* was a salient variable: ‘But I'm happy here…Nobody's trying to kill you, no shoot, no bombs, no suicidal (types), no blasts, nothing’ (RA, M).

Wellbeing was achieved by *adaptation and success* in the new culture. In the resettlement phase, this was achieved by acquiring basic skills (for some, learning English), earning and saving enough money to feel secure, and navigating the system: ‘My husband got a job and now I have a job. Our mental health stress is much less…now we have a fixed plan, a long‐term perspective for our future…we should save money’ (RA, W). Psychological needs that transcended material security included autonomy, reflected in self‐sufficiency: ‘They try their best to provide and reach self‐sufficiency. They come up with a down payment and go get their own home and regularly pay mortgage and they are responsible’ (CE/RA, M).

Goals include *functionality in essential roles*, including gender roles: ‘For women I think it's being the homemaker and taking care of their family, supporting their children in their education goals. For men, it's being a provider’ (CE, W); and those related to the connection and obligation to family: ‘I work to support my family back home in Afghanistan, and that's the good lifestyle that I can make enough money to support them’ (CE/R, W); ‘Children are your insurance plan’ (CE/RA, W); ‘I wish that it would be possible for my father and brother to join us here in the U.S…I pray to God that will happen’ (RA, M).

As with most immigrant groups, the change in cultural values creates the need to *manage cultural conflicts*. The possibility of empowerment caused a shift in expectations for girls and women, who, with more autonomy, were willing to seek education, independence, and even divorce: ‘(Women) want to be independent. They want to work. They want to study’ (R/CE, W); ‘I want my daughters get educated here and this is the only thing that I want for my family's future’ (R, W); ‘I went through a lot…and finally I said no…now I'm a single mom and I'm happy…you just have to…think strong’ (CE/RA, W).

### 3.5 Attitudes Towards Host Culture Treatment

Attitudes towards seeking host culture treatment varied by host culture exposure: ‘The Afghan American community that's been here for a couple decades has made progress, but it may take some time for that same level of progress to be seen with newcomers who don't yet have that intersection of Afghan and American culture’ (CE/RA, W).

Nevertheless, there was a dominant belief that ‘treatment’ is for physical illness: ‘They don't have a headache or they don't have pain in the body or stomach…they think that they're healthy. They don't think that mental health, it is a problem’ (CE/RA, W).

Those who did receive formal mental health services had severe symptoms of trauma that interfered with daily function or presented frightening physical symptoms. Represented among our participants were individuals who had found medication and psychotherapy acceptable, especially those who promote strength‐based views: ‘So (doctors) say you have to help yourself…(medicine) will work, but you have to make yourself stronger…one of the doctor says you have to…go outside…breathe well’ (RA, M).

Internal and external stigma included treatment being reserved for serious mental illness, with some shame attached to seeking it: ‘There's a perception that being involved with mental health means that you're extremely mentally where someone may need to be hospitalized or is having a psychotic episode’ (CE, W); ‘Traditionally, I don't go to a doctor and say hey I am worried about this thing in my family…That's kind of a shame in Afghanistan’ (CE/RA, M).

Seeking treatment was also viewed as self‐centred: ‘A lot of families think these groups and these slow activities of worrying about mental health are a luxury that they don't have right now…There's a tendency to view mental health as something for privileged Americans’ (CE, W).

Some noted that sharing emotions, even in safe spaces, is draining: ‘Everyone is out of tears…out of patience for these conversations or maybe even for some of these emotions…they just wanna know, when am I going to learn the language,…get a job, how's my kid gonna do in school’ (CE/RA, W). Other identified barriers included language concerns, discomfort with a stranger, and financial concerns.

Treatment seeking and engagement with talk therapies are not likely to reach the large portion of the population that could use support: ‘Unfortunately, not a lot of people seek out mental health assistance…it's more like reaching out to these people because we realize that they have had trauma’ (CE/RA, W); ‘Things we use here to help people with mental health, Afghans might tend to think that these are not gonna work’ (CE/RA, W).

In summary, Figure 1 describes the components of the RA model as we found them. This very heterogeneous group perceives their difficulties as natural reactions to life circumstances. They report significant emotional symptoms related to flight, which often manifest indirectly. Stressors are caused by environmental conditions, concern about the safety of family members left behind, loss of self and adaptation demands. Natural coping strategies are to avoid, focus on competence, lean on Allah, family, and community, and help others. Perceptions of wellbeing include safety, prosperity and fulfilling valued familial and societal roles, rather than self‐focused (e.g., personal happiness). Cultural conflict is seen as a threat. Population‐specific and contextual nuances include significant guilt for endangering family members left behind, a focus on concrete solutions to manage environmental frustrations, minimal value seen in exploring emotions and adjustment to a greater possibility for the empowerment of women and girls.

## 4 Discussion

Our study extends the literature by giving voice to the experience of individuals themselves and by focusing on the latest, concentrated population of individuals fleeing Afghanistan following the 2021 regime change. Many themes that emerged align with a recent review of Afghan mental health (Alemi et al. 2023), including somatic complaints, the importance of family and faith and distress concerning financial instability, loss of social status and gender role changes. Our study highlights the nuances of this refugee population's shared experiences, given the nature of their flight and post‐migration stressors attributed to suffering, providing a context that might be useful to clinicians. In addition, our study advances the field of international psychology by modelling the use of explanatory frameworks to bridge the gap between health beliefs of host culture practitioners and patients from diverse cultures.

Our findings align with those of Lavdas et al. (2023), who identified current conditions as a frequent causal attribution for depression among Afghan refugees in Greece. They also reported that women attributed distress to gender‐based and domestic violence and sought interpersonal support to cope, whereas men attributed distress to conflict and persecution and utilised self‐empowerment and solution‐oriented coping. Our study adds to this literature by capturing some of the tensions that occur when women and girls try to break out of these gender roles in the host culture. Our study found that avoidance, social activation and religion are natural coping skills, which align with strategies for managing distress identified in a population of Afghan refugees in New Zealand and Australia (e.g., exercising, socialisation, avoidance, relaxation and religion) (Sulaiman‐Hill and Thompson 2012). Our findings extend the literature by highlighting the importance of achieving competence and helping other refugees in this population.

Our study elucidates significant post‐migration distress associated with experiencing the sudden 2021 Taliban takeover. While Lavdas et al. (2023) illustrated post‐migration stress associated with witnessing the 2021 Taliban takeover through social media, their sample lacked participants who directly experienced the regime change and migrated as a result. The ongoing instability, violence and threats to safety since this regime change are not unfamiliar to Afghans, but were exacerbated by the simultaneous sudden withdrawal of international forces, wreaking havoc on social support systems and threatening psychological resilience (Tay 2024). This population of Afghan refugees includes many whose work for the US armed forces in Afghanistan provided them an opportunity to enter their host country, yet created danger for their families now targeted for this connection to US forces. Such factors have pronounced effects on mental health within this population, including fear for family members left behind. These contextual nuances might indicate a need for mental health interventions that address survivors' guilt and grief among this population. These findings and implications expand upon a recent quantitative study of this particular population of Afghan refugees in the United States, which found that women, minoritised ethnic groups, those who experienced prolonged displacement, and refugees with uncertain visa statuses were at higher risk for collective trauma and poor mental health outcomes, emphasising the need for tailored interventions that address the needs of these at‐risk individuals (Kirsch et al. 2024).

Our results gave us insight into the discrepancies between the standard host culture treatment model and Afghan health beliefs outlined in Table 1. In searching for solutions to address discrepant health models, we examined coping strategies of those who seemed to be thriving and identified the following confidence enhancers: make stepwise plans, take small risks, aspire to regain former identity/status, take opportunities to grow desired skills (e.g., language) and engage community role models who can offer personal anecdotes and encouragement. With these in mind, we propose clinical recommendations that aim to meet clients where they are while applying clinical skills consistent with their values to move them forward.

**TABLE 1:** Recommended clinical approaches informed by discrepancies between participant and host culture explanatory models of mental illness/health.

Explanatory domain	Host culture model	Participant model	Recommended clinical approaches
Experiences of distress	Symptoms indicate a disorder	Emotional distress is part of life	Respect and understand the client's identities and experiences
Causal attributions for suffering	Suffering is caused by demands overwhelming resources	Suffering is caused by environmental events	Follow client's lead Offer solution‐focused services
Coping strategies	Aims to teach skills to manage symptoms Involves talking about emotions and cognitions Relies on expert practitioners to alleviate distress Medication supports overwhelmed resources	Seeks skills to manage the environment Natural management style is to avoid emotions Relies on Allah, family, community Medication is for physical illness	Coordinate collaborative care across disciplines Strengthen natural coping Destigmatise help‐seeking Build bridges
Goals for wellbeing	Seeks ‘understanding’ of emotions Aims to treat suffering	Seeks resolution of stressors Suffering is not a target of treatment	Focus on resettlement Link treatment strategies to client's goals

## 5 Recommended Clinical Approaches

Respect for the client's goals involve prioritising skills to facilitate basic adaptation. Delivering resources might be an important first step. Recognising stigma and respecting the strategies used to meet these goals is equally important. Treatment options might be destigmatised by normalising experiences, emphasising prevention and skills‐based approaches, and offering services to all. Integrating offerings with other skill‐based social services and healthcare might reduce the burden of separate appointments on overwhelmed clients. Clinicians are also encouraged to actively reflect on and develop their own cultural competence and practice cultural humility. Such an approach is consistent with strengthening their clients' natural coping strategies. Our study suggests that these include developing a stepwise plan to achieve goals, supporting optimism, promoting social connections within their community, partnering with cultural and religious experts and bringing in role models of Afghan background to share their stories and offer motivation.

We recommend that clinicians start by planting seeds. Many new arrivals are overwhelmed with meeting basic survival needs and may not be open to receiving services viewed as non‐essential. Coordinating treatment with non‐psychiatric components of care (e.g., social work) is essential. Clinicians can stay available and build bridges by linking the strategies they have to offer to the client's goals (e.g., the possibility for improving sleep to facilitate effective adaptation). Solution‐focused therapy that aligns with more concrete problems and goals (de Shazer et al. 1986) might be most acceptable. In appropriate cases, the culturally congruent belief that physical symptoms such as sleep and appetite changes can be treated with medication can help build the bridge to psychiatry consultation. Care offered in interdisciplinary teams that work collaboratively to support clients' intertwined needs would be the most acceptable and effective means to provide psychological services to this population.

As clinicians engage their clients in therapy, trust can be built by being upfront about the boundaries of the services offered while facilitating referral to appropriate support. This requires a conversation about the value of clinical services and transparency around what a therapist cannot accomplish (e.g., help with funds and immigration issues). Clinicians should consider how therapeutic techniques can be integrated into their value system in nontraditional formats. For example, one CE indicated success with a woman who viewed self‐care as ‘selfish’ by exploring how she might incorporate mindfulness into valued activities such as cooking or cleaning. Alternative methods of delivering brief mental health interventions, such as via peer refugee helpers, could align with altruism goals and support an established cultural preference to rely on peer networks for mental health needs (Eggerman and Panter‐Brick 2010). Such peer‐based interventions have demonstrated acceptability, feasibility and efficacy among Syrian refugees in the Netherlands (de Graaff et al. 2020).

It is well to be informed and respectful of gender roles and standards, which differ by household. There are layers behind the initial view of women being entirely subservient to men. More rigid gender roles are particularly relevant to new arrivals, in contrast to earlier arrivals, whose extended host culture exposure might promote their acceptance of more flexible gender norms. In order to reach Afghan refugee women who migrated after 2021, clinicians might have to make contact through their husbands and should expect that women may not speak openly in front of men. The empowerment of women is a new possibility for many. Parents are required to accept more equal gender roles for the younger generation with mandatory education. Subtle cultural shifts occur that increase autonomy, as many parents find themselves relying on and benefiting from their daughters' acquisition of English and cultural negotiation skills. These changes also affect men in the household, who may require support when deprived of their traditional role as the provider (van de Put 2002). Clinicians ought to be mindful of the nuanced experiences of Afghan refugee women, many of whom have experienced gender‐based violence pre‐ and post‐migration, which might influence their beliefs about mental health experiences (Lavdas et al. 2023).

Finally, the challenge to develop rapport across the cultural divide might be promoted by respect for the client's identities and experiences. Acknowledgement of the client's past status is a strategy to communicate respect. In Afghanistan, men of professional and otherwise high status would be referred to by their profession and an honorific (e.g., Doctor *sahib* or Engineer *sahib*). A culturally similar individual, such as an interpreter, might provide the appropriate name to signal respect. One of our cultural experts made a plea to clinicians on behalf of her community: what appears as demanding or ungrateful behaviour reflects despair and desperation. Practising patience and empathising with the frustrations that result from clients' difficult life experiences can promote a strong therapeutic relationship.

## 6 Limitations and Future Directions

This study examined a heterogeneous group of refugees from Afghanistan displaced by the 2021 regime change, settled in an urban area of the United States that has a relatively large Afghan refugee community. Findings might not generalise to those resettled in more rural, less diverse settings, or to locations outside the southwestern US and overseas. Despite this limitation, our discrepancy model more broadly serves as an example of how to bridge culture‐specific mental health beliefs of diverse populations with practitioners across the globe for understanding mental health, illness and treatment. Recommendations must be further adapted for clinicians outside the United States based on their culturally informed model of care.

Cultural communication norms which emphasise turning to family and community might have discouraged participants from sharing their mental health struggles with strangers on the research team. Findings may underestimate the prevalence and/or severity of symptoms among this population. We also acknowledge language as a barrier to communication among non‐English speaking participants. Finally, we recognise that the researcher and interviewer positionality may have affected the selection of results and the interpretation of data. Despite these limitations, an important strength of this study was its collaborative approach to deriving themes, including and prioritising the perspective of study team members who originated from Afghanistan.

Ultimately, broad statements about the perceptions and needs of any group would require the inclusion of many more participants and explorations of moderators based on demographic and sub‐culture variation. Our results might anchor future studies which investigate mental health symptoms and disorders, identify salient risk and protective factors, and inform the development of culturally tailored interventions. This study contributes to the field by elucidating the mental health beliefs and experiences of Afghan refugees in the United States who were displaced following the 2021 Taliban takeover and provides practical suggestions for clinicians managing this population. We hope this example of bridging client and clinician explanatory models might offer tools to develop rapport in the therapeutic relationship, allow for cultural adaptation, and increase acceptability of treatments offered.

## Author Contributions

**Savannah Dieste:** conceptualization, methodology, project administration, investigation, data curation, resources, visualization, formal analysis, writing – original draft, writing – review and editing. **Freschta Naseri:** conceptualization, investigation, formal analysis, writing – review and editing. **John Dykema:** conceptualization, resources, formal analysis, writing – review and editing, supervision. **Chaundra Merrell:** conceptualization, resources, project administration, formal analysis, writing – review and editing. **Alexander Laywell:** conceptualization, resources, project administration, formal analysis, resources, writing – review and editing. **Hamayoun Kargar:** conceptualization, investigation, formal analysis, writing – review and editing. **Sunita M. Stewart:** funding acquisition, project administration, investigation, conceptualization, methodology, formal analysis, writing – original draft, writing – review and editing, supervision.

## Disclosure

The authors have nothing to report.

## Ethics Statement

This study was approved by the [anonymised for review] Institutional Review Board and performed in accordance with the ethical standards as laid down in the 1964 Declaration of Helsinki and its later amendments.

## Consent

Informed consent was obtained from all individual adult participants included in the study.

## Conflicts of Interest

The authors declare no conflicts of interest.

## Endnotes

^1^
Like others (Khan et al. 2022), we recognise the dichotomy of our use of the term ‘Global North’ renders superficial the variability across cultures, but selected this term for succinct communication with the least potential for negative bias.
